# Dietary Behavior and Depressive Symptoms in Late-Life Marriage

**DOI:** 10.1093/geroni/igaa057.1190

**Published:** 2020-12-16

**Authors:** Talha Ali, Gail McAvay, Joan Monin

**Affiliations:** 1 Yale University, New Haven, Connecticut, United States; 2 Yale School of Medicine, New Haven, Connecticut, United States

## Abstract

Epidemiologic studies have linked dietary patterns to psychological health including depression, anxiety, and stress. However, no research has examined dyadic associations between dietary behavior and depressive symptoms in older married couples. In this study, one hundred and one couples 51 to 90 years of age who were married or in a marriage-like relationship and living together for at least 6 months were recruited. Participants completed questionnaires and self-reported their dietary behavior (i.e., the total number of meals, number of snacks, and number of fast-food meals eaten in a typical day and the number of meals they eat alone and eat sitting down). They also completed the 20-item Center for Epidemiologic Studies Depression Scale. Results of the Actor Partner Interdependence Models controlling for income, education, chronic conditions, and marital satisfaction, showed that for wives only, more meals eaten in a day were associated with lower depressive symptoms (actor effect). Additionally, more snacks eaten by the wife and more meals eaten alone by the wife were associated with higher depressive symptoms for the husband (partner effects). Findings suggest that wives’ dietary behavior is particularly important, not only for their own but also their husbands’ mental health in late-life marriage.

